# Oral Soft Tissue and Jawbone Sarcomas: A Retrospective Clinicopathologic Analysis of 128 Cases from Two Institutions and Comprehensive Literature Review

**DOI:** 10.1007/s12105-025-01811-0

**Published:** 2025-06-17

**Authors:** Prokopios P. Argyris, Gabrielle R. Dennis, Rajaram Gopalakrishnan, Ioannis G. Koutlas, Kristin K. McNamara, John R. Kalmar

**Affiliations:** 1 Division of Oral and Maxillofacial Pathology, The Ohio State University College of Dentistry, Postle Hall, Room 2191, 305 W. 12th Avenue, Columbus, OH 43210 USA; 2 Department of Oral and Maxillofacial Surgery, Loma Linda University School of Dentistry, Loma Linda, CA USA; 3 Division of Oral and Maxillofacial Pathology, School of Dentistry, University of Minnesota, Minneapolis, MN USA

**Keywords:** Oral soft tissue sarcoma, Oral cavity sarcoma, Jawbone sarcoma, Oral soft tissue and bone malignancies, Oral mesenchymal neoplasms, Osteosarcoma, Kaposi sarcoma

## Abstract

**Purpose:**

Oral soft tissue and jawbone sarcomas (OSTJS) are rare neoplasms accounting for only 1% of all intraoral malignancies. As a result, robust epidemiologic data pertaining to OSTJS are limited. Here, we present a collaborative, retrospective analysis of the clinicopathologic characteristics of 128 cases of OSTJS, together with a comprehensive review of the literature.

**Methods:**

Archived OSTJS cases (2000–2022) were retrieved from the electronic laboratory databases of the oral pathology services at The Ohio State University and University of Minnesota. Patient age and sex, anatomic site and histopathologic diagnosis were recorded.

**Results:**

Among 128 OSTJS, 123 (96.1%) were primary and 5 (3.9%) metastatic (M: F = 1.5:1; mean age = 43.7 years, range = 4-102 years). Most OSTJS presented in adults (113, 88.3%; mean age = 47.8 years) with only 15 pediatric cases (11.7%; mean age = 13 years). Favored sites included the mandible (48, 37.5%), maxilla (39, 30.4%), gingiva (15, 11.7%), palate (13, 10.2%), and tongue (4, 3.1%). In adults, osteosarcoma represented the predominant OSTJS (58, 51.3%), followed by Kaposi sarcoma (18, 15.9%), leiomyosarcoma (7, 6.2%), chondrosarcoma (6, 5.3%), low-grade myofibroblastic sarcoma (5, 4.4%), and 4 each (3.5%) of angiosarcoma, rhabdomyosarcoma and undifferentiated pleomorphic sarcoma. Similarly, osteosarcoma comprised the most common OSTJS histotype in the pediatric population (8, 53.3%), followed by Ewing sarcoma (4, 26.7%) and 1 each (6.7%) of *TFCP2::EWSR1*-rearranged rhabdomyosarcoma, mesenchymal chondrosarcoma, and alveolar soft part sarcoma.

**Conclusion:**

OSTJS represent an uncommon, histopathologically diverse, subset of mesenchymal malignancies. In our series, most patients were adults in their 4th − 5th decade with a broad age range and a slight male predilection. Overall, jawbone osteosarcoma and Kaposi sarcoma accounted for two-thirds of OSTJS cases in this cohort. While the diagnosis of OSTJS relies heavily on routine light microscopic findings, ancillary immunohistochemistry and/or cytogenetic studies are frequently warranted.

## Introduction

Sarcomas arising in the soft tissues and bone represent a relatively rare group of mesenchymal neoplasms comprising 15 − 21% of pediatric and 1% of adult solid malignancies [1–3]. According to the Surveillance, Epidemiology, and Ends Results Program of the National Cancer Institute (SEER), the annual incidence of soft tissue sarcomas (STS) is approximately 3.4 cases per 100,000 with 13,520 new diagnoses predicted in the United States in 2025, and nearly 5,420 individuals anticipated to succumb to the disease [4]. Although anatomically ubiquitous, STS show a strong predilection for the lower and upper extremities, chiefly the thigh, collectively encompassing 40 − 45% of all reported cases, while other sites of involvement may include the viscera (22%), retroperitoneum (16%) and trunk (10%) [1, 5].

Bone and STS of the head and neck account for 6 − 15% [6, 7] of all sarcomas affecting adults but merely 1 − 2% of head and neck malignancies [7–9], whereas during childhood and adolescence head and neck STS comprise approximately one-third of all pediatric sarcomas and 12% of head and neck cancers [6, 10]. Clinically, such lesions present primarily as asymptomatic masses of variable size and growth rate [11–13]. Depending on the anatomic structures involved, accompanying findings may include prolonged epistaxis or nasal discharge, proptosis, visual impairment, otalgia, and sensory and/or motor disturbances. Pain and paresthesia, when reported, are usually associated with intraosseous lesions, e.g., osteosarcoma, chondrosarcoma or Ewing sarcoma [11]. Additionally, osteosarcomas of the jawbones frequently present with marked cortical expansion, tooth mobility with symmetrical widening of the periodontal ligament space, and root resorption [14, 15].

In contrast to the striking majority of head and neck squamous cell carcinomas (SCC), tobacco and alcohol consumption does not appear to participate in sarcomagenesis [16]. Although most bone and STS of the head and neck represent sporadic (idiopathic) tumors, well-documented predisposing factors include radiation therapy, viral infections, e.g., HIV and HHV-8 (KSHV), and inherited syndromic conditions with increased predisposition to mesenchymal malignancies, such as Li-Fraumeni, familial retinoblastoma, Bloom, Werner, Rothmund-Thomson and neurofibromatosis type I [1, 3]. Notwithstanding modern multimodality therapeutic regimens, prognosis for patients with head and neck STS is dismal with reported overall 5-year survival rates ranging between 46% and ~ 66% [7, 8, 11, 17]. Outcomes are dependent on both clinical, i.e., location, size, presence of distant metastasis, and primary tumor resectability, as well as histopathologic characteristics including histologic subtype and tumor grade [7–9, 11, 16, 18, 19]. However, consensus among various cohorts regarding the prognostic utility of the above factors has yet to be achieved.

Oral soft tissue and jawbone sarcomas (OSTJS) are rare, encompassing 10% and 4% of head and neck sarcomas in adults and children, respectively [7]. Owing to their rarity, robust epidemiologic data pertaining to the incidence of OSTJS are limited. Furthermore, adding to the vexing task of assessing OSTJS frequency, such lesions are commonly lumped and reported together with extraoral, head and neck bone and STS [7, 9, 17]. Consequently, clinicopathologic studies focusing on OSTJS proper are sparse in the English literature [20–28].

Herein, we present a large-scale, collaborative, retrospective analysis of the epidemiologic and clinicopathologic characteristics of OSTJS, together with a comprehensive review of the pertinent literature.

## Materials and Methods

### Case Identification and Selection

Following IRB approval (Study Number: 2022C0194), the electronic databases of the Oral Pathology Laboratory at the School of Dentistry, University of Minnesota, and Oral Pathology Consultants at The Ohio State University, College of Dentistry were searched for archived cases of sarcoma diagnosed during the period 2000–2022. Inclusion criteria comprised: (i) primary or metastatic mesenchymal malignancies involving the soft tissues of the oral cavity or gnathic bones, and (ii) adequate confirmation of the diagnosis based on reported histomorphologic, immunohistochemical and/or molecular findings. Primary cutaneous or sinonasal tumors with intraoral involvement were excluded from this study, as were cases with incomplete histopathologic documentation. Examples of sarcomatoid (spindle cell) SCC and myeloid sarcoma were also excluded. Patient demographics, i.e., age and sex, lesion anatomic site, and histopathologic diagnosis were recorded.

### Literature Review on OSTJS

Publicly available electronic databases, including PubMed, MedScape, and Google Scholar, were searched for previously reported case series of OSTJS published in the English literature during the period 1995–2025. The following keyword combinations were used: “oral sarcoma”, “oral soft tissue sarcoma”, “sarcoma of the oral cavity”, “jawbone sarcoma”, and “gnathic sarcoma”. Single case reports and case studies confined to a specific histopathologic subtype were excluded from the present literature review. Furthermore, published series of head and neck bone and STS lumping together cases from both intra- and extraoral locations were deemed ineligible for inclusion.

Only nine previous reports satisfying the inclusion criteria were identified and comprised 7 full articles [20–22, 24–26, 28], 1 short communication [23], and 1 published abstract [27]. Available information regarding patient sex, age mean and range, as well as lesion location and histopathologic subtype was recorded and utilized for further analysis (Table 1). Upon careful evaluation of the list of histopathologic diagnoses provided in the above studies, lesions diagnosed as plasmacytoma [22], myeloid sarcoma [25], histiocytic sarcoma [28], dendritic cell sarcoma [28], and dermatofibrosarcoma [26] were excluded from the current literature review of OSTJS, similar to cases occurring in extraoral anatomic sites, e.g., facial skin [21], parotid [21], and neck region [24].

**Table 1:** Collective presentation of the clinico-epidemiologic and histopathologic characteristics of previously reported oral soft tissue and jawbone sarcomas (OSTJS) included in the current literature review (*N* = 479)

Authors	Number of cases	Age mean (years; range)	Sex	Location	Histopathologic Diagnosis
Gorsky and Epstein [20]	16	39.2 (3–75)	10 M:6 F	7 Oral cavity, NOS 3 Tongue 2 Palate 1 Gingiva 1 Retromolar pad 1 Buccal mucosa 1 FOM	7 Sarcoma, NOS 4 Rhabdomyosarcoma 2 Leiomyosarcoma 1 Malignant solitary fibrous tumor 1 Fibrosarcoma 1 Carcinosarcoma
Pandey et al. [21]	8	28.9 (15–54)	7 M:1 F	3 Buccal mucosa 2 Tongue 2 Mandibular alveolus 1 Maxilla	3 Rhabdomyosarcoma 2 Spindle cell sarcoma 1 Angiosarcoma 1 Hemangioendothelioma 1 UPS
Yamaguchi et al. [22]	30	41.5 (0.4–77)	22 M:8 F	12 Maxilla/Maxillary sinus 12 Mandible 3 Buccal mucosa 2 TMD fossa 1 Submandibular region	9 Osteosarcoma 7 UPS 5 Rhabdomyosarcoma 3 Fibrosarcoma 2 Leiomyosarcoma 2 Angiosarcoma 1 Liposarcoma 1 Ameloblastic fibrosarcoma
Chidzonga and Mahomva [23]	88	26 ^*^	51 M:37 F	46 Mandible, NOS 42 Maxilla, NOS	34 Osteosarcoma 21 Rhabdomyosarcoma 11 Fibrosarcoma 6 Leiomyosarcoma 5 Chondrosarcoma 2 Mesenchymal chondrosarcoma 2 MPNST 2 Liposarcoma 2 Synovial sarcoma 1 Fibromyxosarcoma 1 Myxosarcoma 1 Ameloblastic fibrosarcoma
Sumida et al. [24]	18	50.5 (17–80)	10 M:8 F	10 Mandible 6 Maxilla/Maxillary sinus 2 Buccal mucosa	6 Osteosarcoma 3 Leiomyosarcoma 3 UPS 2 Rhabdomyosarcoma 1 Angiosarcoma 1 Ewing sarcoma 1 MPNST 1 Undifferentiated sarcoma, NOS
Kumar et al. [25]	24	31.3 (4–75)	17 M:7 F	12 Maxilla 9 Mandible 1 Buccal vestibule 1 Gingiva 1 Buccal mucosa	7 Osteosarcoma 5 Ewing Sarcoma 3 Mesenchymal chondrosarcoma 3 Leiomyosarcoma 2 MPNST 2 UPS 2 Rhabdomyosarcoma
de Carvalho et al. [26]	199	32.2 (3–87) ^**^	112 M:87 F	90 Mandible 41 Oral cavity, NOS 30 Palate 24 Maxilla 14 Gingiva	74 Osteosarcoma 52 Kaposi sarcoma 17 Chondrosarcoma 12 Leiomyosarcoma 7 Rhabdomyosarcoma 6 Fibrosarcoma 5 Synovial sarcoma 5 Ewing sarcoma 4 Liposarcoma 4 Ameloblastic fibrosarcoma 3 Pleomorphic sarcoma 2 Angiosarcoma 2 Spindle cell sarcoma 2 Myxoid sarcoma 1 Alveolar soft part sarcoma 1 MPNST 1 Metastatic osteosarcoma 1 Metastatic PNET
Bisio et al. [27]	22	40 (24–55)	19 M:3 F	12 Palate 10 Oral cavity, NOS	13 Kaposi sarcoma 3 Leiomyosarcoma 2 Fibrosarcoma 1 Alveolar rhabdomyosarcoma 1 UPS 1 Synovial sarcoma 1 Inflammatory myofibroblastic sarcoma
Pina et al. [28]	74	33.9 (0–71) ^**^	45 M:29 F	24 Palate 16 Oral cavity, NOS ^***^ 11 Gingiva 9 Buccal mucosa 8 Tongue 6 Alveolar ridge	26 Kaposi sarcoma 13 Leiomyosarcoma 10 Rhabdomyosarcoma 5 MPNST 4 Liposarcoma 4 Myxoid sarcoma 2 Synovial sarcoma 2 High-grade sarcoma, NOS 2 Spindle cell sarcoma 2 Alveolar soft part sarcoma 1 Carcinosarcoma 1 Undifferentiated sarcoma, NOS 1 Pleomorphic sarcoma 1 Myofibroblastic sarcoma
Total Number	479	35.9 (0–87)	293 M:186 F (M: F = 1.6:1)	167 (34.9%) Mandible 97 (20.2%) Maxilla 74 (15.4%) Oral cavity, NOS 68 (14.2%) Palate 27 (5.6%) Gingiva 19 (4.0%) Buccal mucosa 13 (2.7%) Tongue 6 (1.3%) Alveolar ridge 2 (0.4%) Mandibular alveolus 2 (0.4%) TMD fossa 1 (0.2%) FOM 1 (0.2%) Retromolar pad 1 (0.2%) Buccal vestibule 1 (0.2%) Submandibular region	130 (27.1%) Osteosarcoma ^ƒ^ 91 (19.0%) Kaposi sarcoma 55 (11.5%) Rhabdomyosarcoma ^#^ 44 (9.2%) Leiomyosarcoma 23 (4.8%) Fibrosarcoma 22 (4.6%) Chondrosarcoma 14 (2.9%) UPS 11 (2.3%) MPNST 11 (2.3%) Liposarcoma 11 (2.3%) Ewing sarcoma 10 (2.1%) Synovial sarcoma 9 (1.9%) (High-grade) Sarcoma, NOS 7 (1.5%) Myxoid sarcoma/Myxosarcoma 6 (1.3%) Angiosarcoma 6 (1.3%) Ameloblastic fibrosarcoma 6 (1.3%) Spindle cell sarcoma 5 (1.0%) Mesenchymal chondrosarcoma 4 (0.8%) Pleomorphic sarcoma 3 (0.6%) Alveolar soft part sarcoma 2 (0.4%) Undifferentiated sarcoma, NOS 2 (0.4%) Carcinosarcoma 1 (0.2%) Myofibroblastic sarcoma 1 (0.2%) Malignant solitary fibrous tumor 1 (0.2%) Hemangioendothelioma 1 (0.2%) Fibromyxosarcoma 1 (0.2%) Metastatic osteosarcoma 1 (0.2%) Metastatic PNET 1 (0.2%) Inflammatory myofibroblastic sarcoma

NOS, not otherwise specified; MPNST, malignant peripheral nerve sheath tumor; UPS, undifferentiated pleomorphic sarcoma; PNET, primitive neuroectodermal tumor; FOM, floor of mouth; TMD fossa, temporomandibular fossa ^*^ Detailed age information for each case was unavailable in this short communication; mean age was calculated utilizing the age mean values provided separately for men and women. ^**^ Since detailed age information was not provided for each case, recalculation of the age mean and range was not feasible after exclusion of lesions diagnosed as histiocytic sarcoma, dendritic cell sarcoma or dermatofibrosarcoma. ^***^ In addition to the anatomic sites reported above, the authors generically mentioned other intraoral locations without, however, specifying corresponding number of cases for each of them. Therefore, for the purpose of analysis, these sites were categorized as “oral cavity, NOS”. ^**ƒ**^ This includes osteosarcoma NOS, as well as osteoblastic and chondroblastic variants. ^**#**^ This includes rhabdomyosarcoma NOS, as well as alveolar, embryonal and pleomorphic variants

## Results

### Retrospective Institutional Cohort

**Fig. 1:**
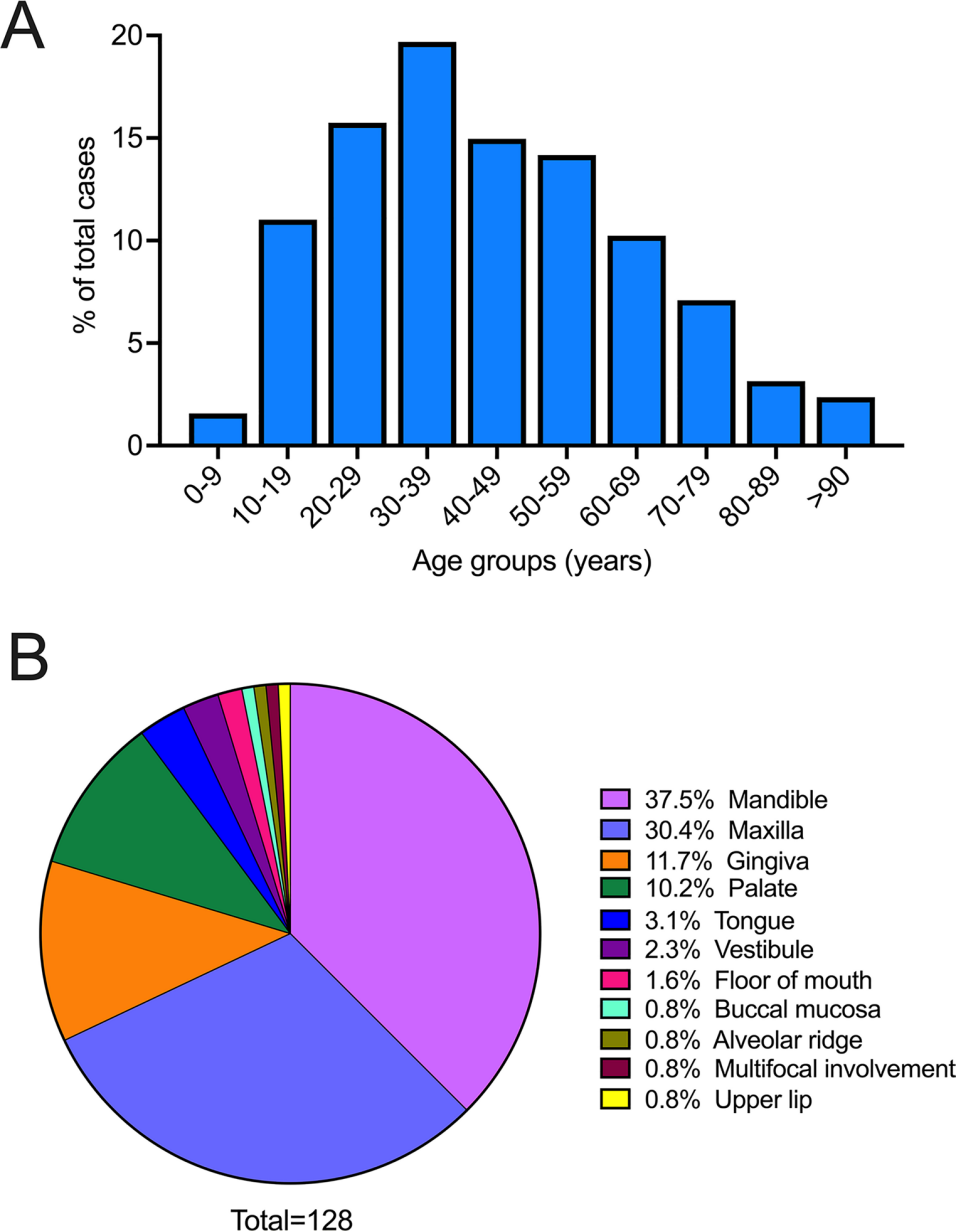
(**A**) Bar graph depicting the age distribution per decade of life and (**B**) pie chart showing the anatomic site distribution of OSTJS (*N* = 128) from the current retrospective study

A total of *N* = 128 OSTJS were diagnosed during this 23-year period, with 123 (96.1%) presenting as primary and only 5 (3.9%) as metastatic lesions. Seventy-seven (60.2%) cases affected men and 51 (39.8%) women (M: F = 1.5:1; age mean = 43.7 years, range = 4–102 years). OSTJS showed a preponderance for adults (113, 88.3%; age group mean = 47.8 years, range = 19–102 years). Notably, approximately half of OSTJS occurred in individuals aged 20–49 years with an apparent peak during the 4th decade of life (Fig. 1A), and only 15 (11.7%) cases involving children and adolescents (age group mean = 13 years, range = 4–18 years). Most frequent anatomic sites included the mandible (48, 37.5%) and maxilla (39, 30.4%), followed by the gingiva (15, 11.7%), palate (13, 10.2%), and tongue (4, 3.1%), while other less common locations comprised the vestibule, floor of mouth, buccal mucosa, alveolar ridge and upper lip collectively accounting for 6.3% of all sites (Fig. 1B). The striking majority of pediatric OSTJS were intraosseous (13, 86.7%) with 9 (60%) occurring in the mandible and 4 (26.6%) in the maxilla, while the remaining 2 cases involved the palate and tongue (6.7% each).

**Fig. 2:**
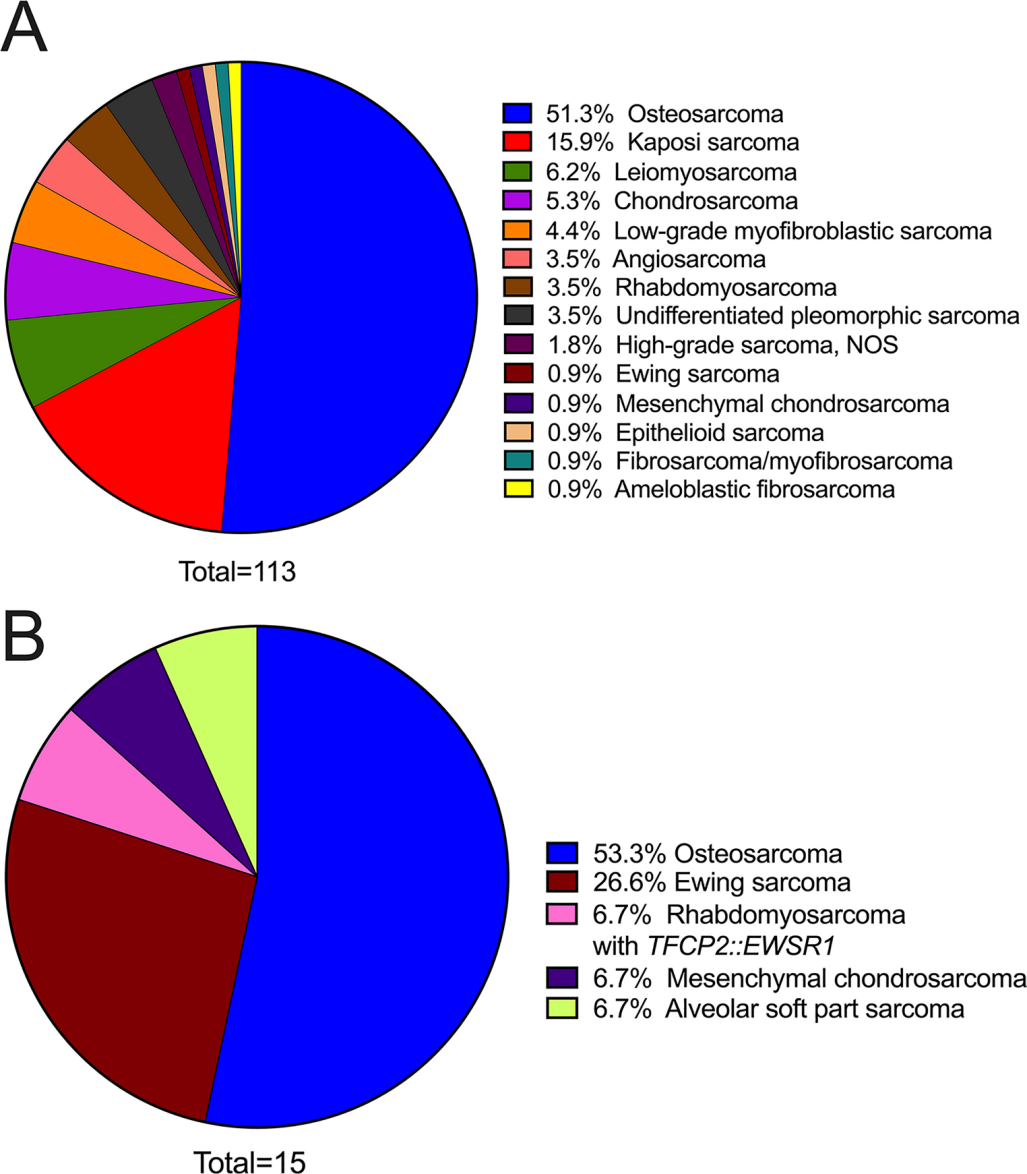
(**A**) Pie chart with the most frequent histopathologic subtypes of OSTJS in the adult, as well as (**B**) pediatric population

**Fig. 3:**
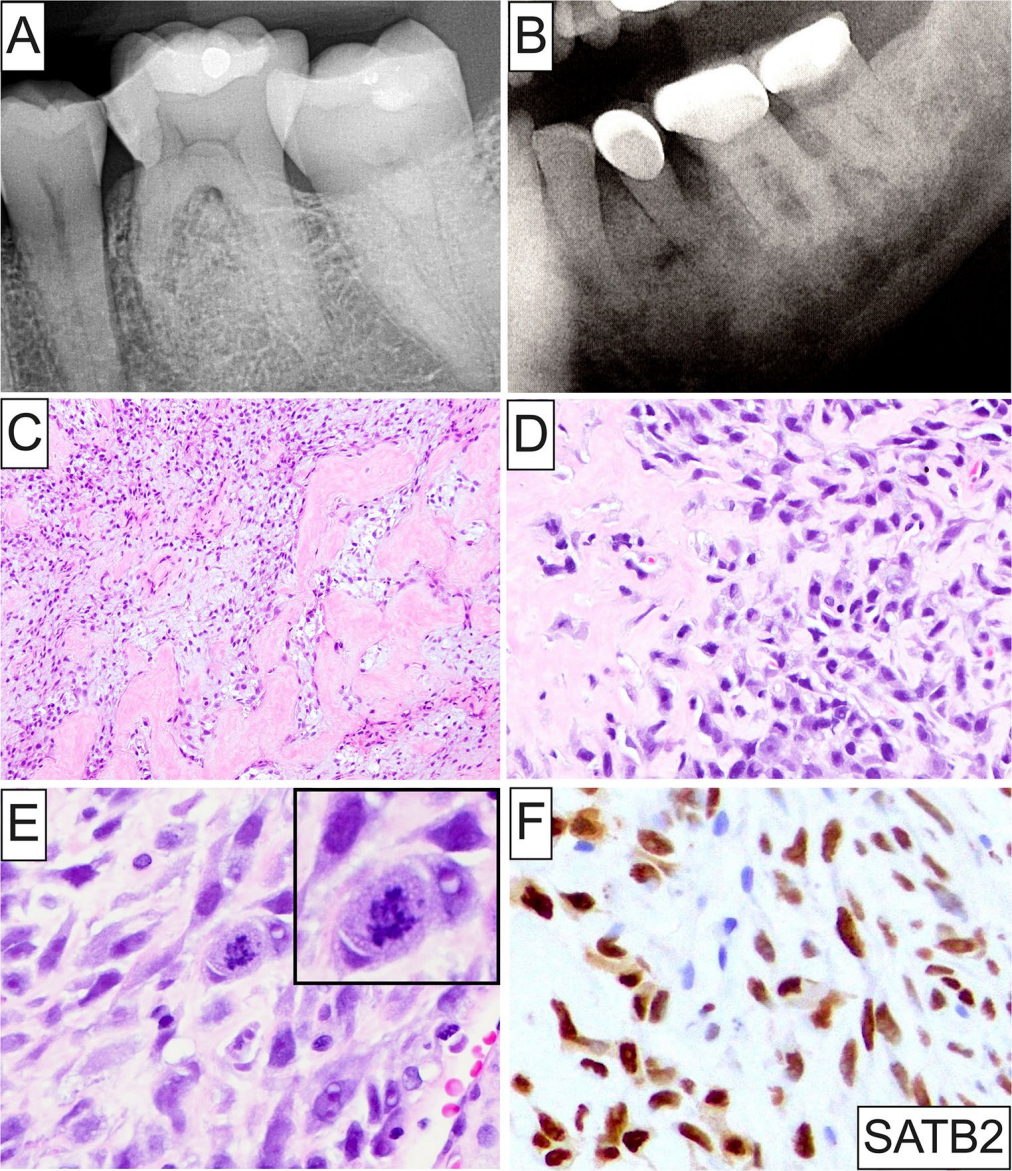
(**A**) and (**B**) Radiographic appearance of mandibular osteosarcomas presenting as poorly-defined, mixed, radiopaque/radiolucent lesions causing widening of the periodontal ligament space and root resorption of involved teeth; (**C**) and (**D**) Low- and medium-power photomicrographs depicting a population of atypical stellate-shaped and spindle cells with hyperchromatic nuclei immersed in a fibromyxoid matrix, in association with eosinophilic osteoid product; (**E**) High-power photomicrograph of osteosarcoma displaying malignant spindle cells exhibiting enlarged, oval or elongated, hyperchromatic nuclei with frequent 1–2 macronucleoli, amphophilic cytoplasm, and irregular mitoses (**inset**); (**F**) Strong and diffuse, nuclear SATB2 immunoexpression is noted in jawbone osteosarcomas

**Fig. 4:**
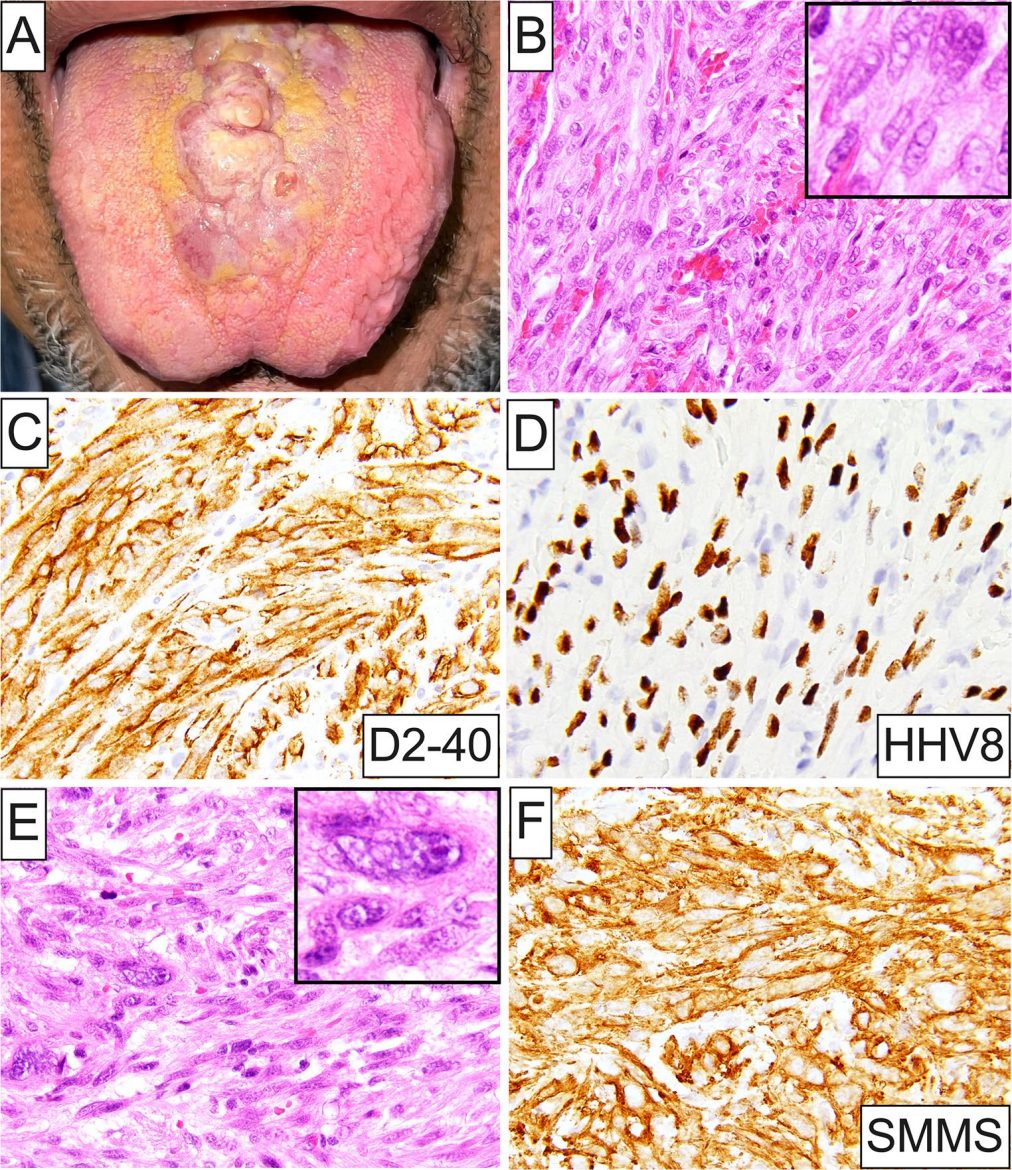
(**A**) Clinical presentation of Kaposi sarcoma involving the dorsal surface of the tongue manifesting as an exophytic, multinodular, erythematous mass (Courtesy of Dr. Alex Daneshgar); (**B**) Histopathologic characteristics of Kaposi sarcoma composed of intersecting fascicles of, overall, bland spindle cells featuring plump, vacuolated nuclei (**inset**) and eosinophilic cytoplasm. Slit-like vascular spaces and copious extravasated erythrocytes are present; (**C**) Kaposi sarcoma cells are diffusely positive for D2-40 (podoplanin), as well as (**D**) HHV8 by immunohistochemistry; (**E**) Histopathologic features of leiomyosarcoma comprising pleomorphic spindle cells arranged in a fascicular growth pattern and characterized by plump, blunt-ended, vesicular nuclei with coarse or finely granular chromatin, and ample eosinophilic cytoplasm. Bizarre, enlarged nuclei with prominent acidophilic nucleoli are also noted (**inset**); (**F**) Leiomyosarcoma showing strong and diffuse positivity with smooth muscle myosin (SMMS) immunostaining

**Fig. 5:**
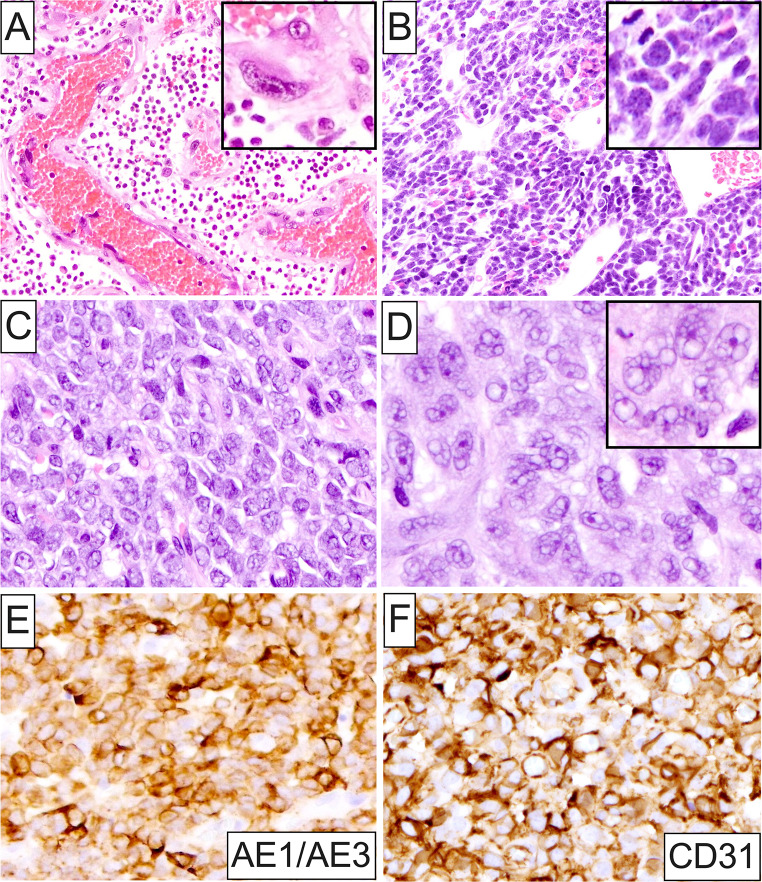
(**A**) Histopathologic characteristics of intraoral angiosarcoma exhibiting numerous, variably-shaped, congested, vascular spaces lined by a single or multiple layers of overtly pleomorphic endothelial cells. Lesional cells feature nucleomegaly with coarse chromatin and occasional macronucleoli (**inset**), together with varying amounts of eosinophilic cytoplasm. (**B**) Mesenchymal chondrosarcoma of the jaws composed of sheets of basaloid cells showing dark-staining nuclei with speckled chromatin, scant eosinophilic or clear cytoplasm, and readily identifiable mitotic figures (**inset**). A hemangiopericytomatous vasculature is observed. Lobules of hyaline cartilage were also identified in different areas of the tumor. (**C**) and (**D**) Histopathologic findings in an example of primary Ewing sarcoma involving the tongue. Sheet-like arrangement of round-to-ovoid malignant cells featuring pleomorphism, high N: C ratio, oval vesicular nuclei with 1–2 acidophilic macronucleoli, and eosinophilic or amphophilic vacuolated cytoplasm (**inset**). Numerous mitoses are also present. Molecular analysis confirmed an underlying *EWSR1::FLI1* fusion. (**E**) Immunophenotypic profile of the Ewing sarcoma case shown in (C) and (D). By immunohistochemistry, this tumor demonstrated aberrant expression of pancytokeratin AE1/AE3, as well as (**F**) CD31

A markedly broad spectrum of OSTJS histopathologic subtypes was identified (Fig. 2A and B). In adults, osteosarcoma represented the predominant OSTJS accounting for greater than half (58, 51.3%) of diagnosed cases in this population (Figs. 2A and 3A-F), followed by Kaposi sarcoma (18, 15.9%; Fig. 4A-D), leiomyosarcoma (7, 6.2%; Fig. 4E and F), chondrosarcoma (6, 5.3%), low-grade myofibroblastic sarcoma (5, 4.4%), and 4 each (3.5%) of angiosarcoma (Fig. 5A), rhabdomyosarcoma and undifferentiated pleomorphic sarcoma (Fig. 2A). Other rare OSTJS variants observed in adults encompassed mesenchymal chondrosarcoma (Fig. 5B), Ewing sarcoma (Fig. 5C-F), epithelioid sarcoma, high-grade fibrosarcoma/myofibrosarcoma, and ameloblastic fibrosarcoma (1 case each, 0.9%; Fig. 2A). Finally, in 2 (1.8%) cases a diagnosis of high-grade sarcoma, not otherwise specified (NOS) was provided. Of note, Kaposi sarcoma was the most common ST, i.e., extragnathic, sarcoma comprising 46.2% (18 of 39) of all STS involving the oral cavity.

**Fig. 6:**
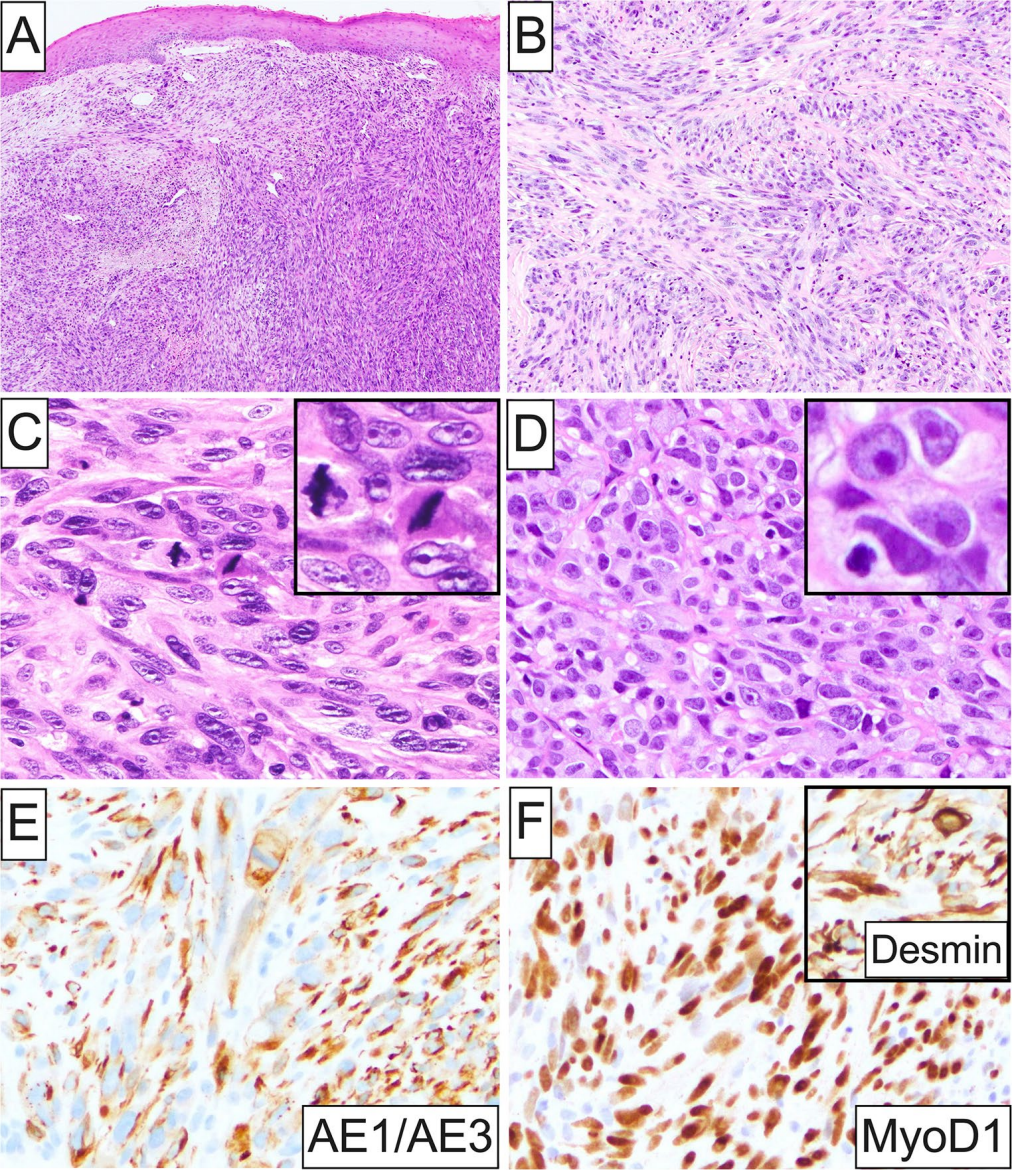
Histopathologic and immunophenotypic characteristics of rhabdomyosarcoma with *TFCP2::EWSR1* rearrangement affecting the posterior mandible of a pediatric patient. (**A**) and (**B**) Low-power photomicrographs depicting a diffusely infiltrative proliferation of predominantly spindle cells organized in intersecting streaming fascicles; the overlying surface epithelium appears intact. (**C**) and (**D**) High-power photomicrographs highlighting the biphasic cytomorphology of this rhabdomyosarcoma variant encompassing highly atypical spindle and epithelioid or round cells featuring marked nuclear pleomorphism, increased N: C ratio, 1 or more prominent acidophilic nucleoli (**insets**), and ample eosinophilic cytoplasm with distinct cell membrane borders. Brisk mitotic activity is also evident (**insets**). (**E**) Malignant cells are strongly and diffusely positive for pancytokeratin AE1/AE3, in addition to (**F**) MyoD1 and desmin (**inset**). A detailed report on this case has been previously published [62]

Interestingly, similar to the adult population, the principal OSTJS histotype in the pediatric age group was osteosarcoma (8, 53.3%; Fig. 2B), followed by Ewing sarcoma (4, 26.7%) and 1 each (6.7%) of rhabdomyosarcoma with *TFCP2::EWSR1* rearrangement (Fig. 6A-F), mesenchymal chondrosarcoma, and alveolar soft part sarcoma.

### Literature Review Analysis on OSTJS

A total of *N* = 479 reported OSTJS cases were identified in the English literature from 9 previously published studies [20–28] using the described inclusion criteria. Available clinico-epidemiologic and histopathologic data pertaining to these cases are summarized in Table 1. Among the 479 OSTJS, 293 (61.2%) affected men and 186 (38.8%) women (M: F ratio = 1.6:1) with a strong predilection for the fourth decade of life (age mean = 35.9 years; age range = 0–87 years). A specific anatomic site, other than “oral cavity, NOS” (74, 15.4%), was provided in 405 cases with the mandible (167, 34.9%) and the maxilla (97, 20.2%) representing the predominant sites of involvement, collectively accounting for greater than 55% of all reported sites. Other locations included the palate (68, 14.2%), gingiva (27, 5.6%), buccal mucosa (19, 4.0%), tongue (13, 2.7%) and alveolar ridge (6, 1.3%).

**Fig. 7:**
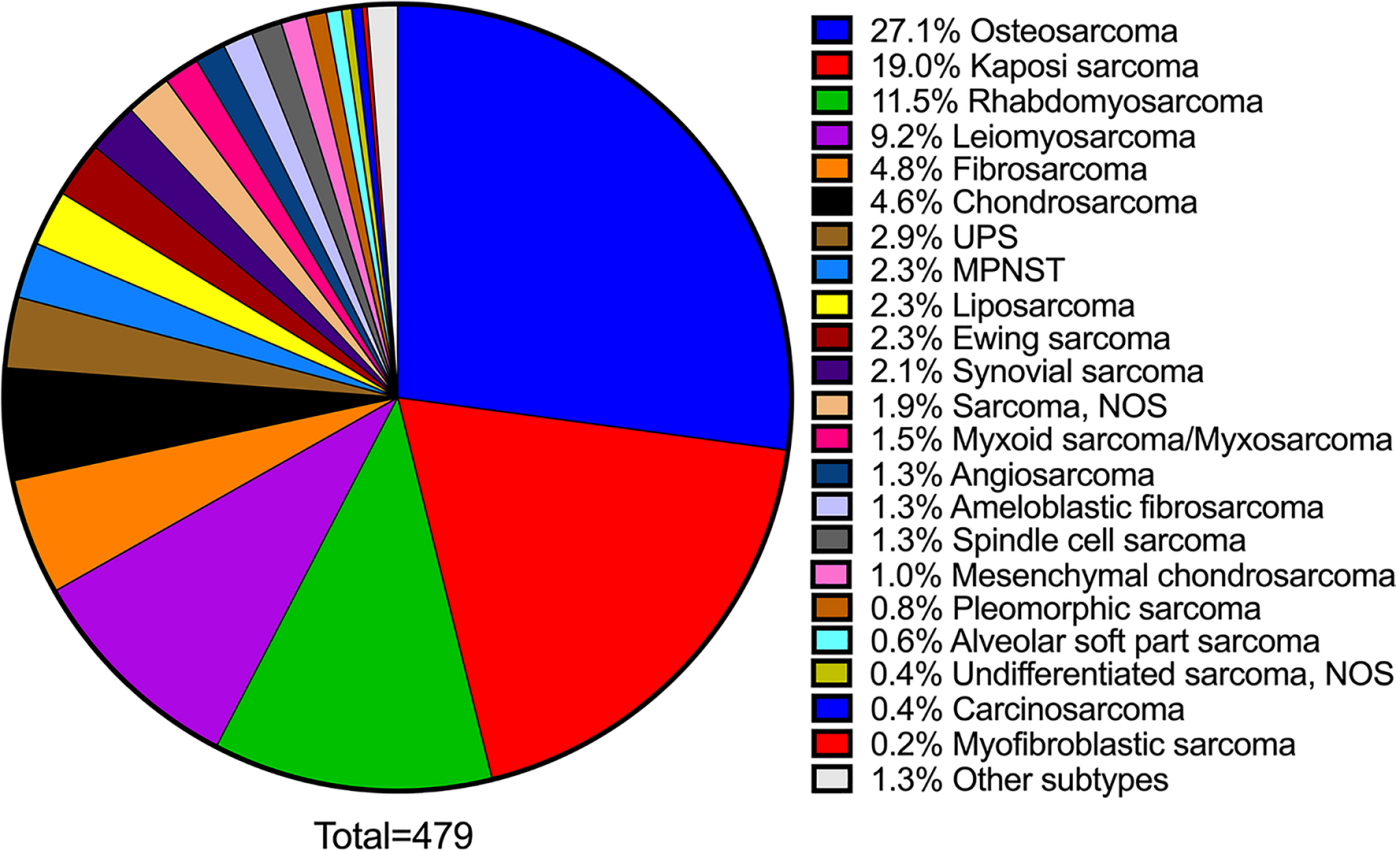
Pie chart depicting the histopathologic repertoire of a total of 479 OSTJS retrieved from the retrospective literature review of 9 previously published studies summarized in Table 1

Mirroring the observed clinical findings, i.e., jawbone involvement encountered more frequently than that of oral soft tissues, osteosarcoma (130, 27.1%) comprised the principal histopathologic subtype of OSTJS (Fig. 7; Table 1), followed by Kaposi sarcoma (91, 19.0%), rhabdomyosarcoma (55, 11.5%), leiomyosarcoma (44, 9.2%), fibrosarcoma (23, 4.8%) and chondrosarcoma (22, 4.6%). Less common, well-recognized, histopathologic variants included undifferentiated pleomorphic sarcoma (UPS; 14, 2.9%), malignant peripheral nerve sheath tumor (MPNST; 11, 2.3%), liposarcoma (11, 2.3%), Ewing sarcoma (11, 2.3%), and synovial sarcoma (10, 2.1%), as well as myxoid sarcoma/myxosarcoma (7, 1.5%), angiosarcoma (6, 1.3%), ameloblastic fibrosarcoma (6, 1.3%), mesenchymal chondrosarcoma (5, 1.0%) and alveolar soft part sarcoma (3, 0.6%). A comprehensive list of all previous OSTJS histopathologic diagnoses included in the present literature review is presented in Table 1 and corresponding Fig. 7.

## Discussion

OSTJS encompass merely 0.1 − 1% of all cancers arising in the oral and maxillofacial region [14, 20, 24–26] and are significantly less common than malignant tumors of surface epithelial origin (including oral SCC and verrucous carcinoma), salivary gland malignancies, and intraoral non-Hodgkin lymphomas [29, 30], with an incidence similar to mucosal melanoma [31–34]. Underscoring the rarity of OSTJS is the scarcity of epidemiologic and clinicopathologic studies on this topic with only 9 well-documented articles identified in the literature [20–28]. Additionally, many of the previously reported OSTJS series are hindered by limited sample size, for instance fewer than 30 cases, and/or lack of stringency pertaining to their histopathologic inclusion criteria. Comparison of the demographic and clinical findings of our institutional case series of *N* = 128 OSTJS to a total of 479 previously published examples summarized in Table 1 reveals striking similarities regarding the age, sex and lesion site distribution. Specifically, in both analyses OSTJS showed a slight male predilection and a preponderance for adults with a mean age approximating 40 years (35.9 and 43.7 years, respectively), and a markedly broad range spanning from the first year(s) of life to the 9th and 10th decades. In contrast, pediatric OSTJS accounted for less than 12% of all tumors in the current study with previous series reporting an estimated incidence of 5.6% [24], 13.3% [22], 29.1% [25] and even as high as 42% [23]. The observed frequency variations may be attributed to sample size discrepancies among studies, geographic and ethnic differences, or institutional referral bias, e.g., pediatric OSTJS patients are more likely to be referred for diagnosis and proper management to a specialized medical center than, for instance, an oral pathology laboratory. Irrespective of age, lesions involving the jawbones distinctly predominated with the mandible and maxilla collectively comprising over half (67.9%) of OSTJS in the present study, a finding further corroborated by the literature review results (55.1%). Additionally, extragnathic (extraosseous) oral STS most frequently occurred in the gingiva and palate with these locations together accounting for ~ 20% of all cases in both the institutional and literature review analyses.

The histopathologic repertoire of bone and STS is markedly broad with more than 50 distinct subtypes recognized in the most recent WHO classification [35]. With the exception of site-specific mesenchymal malignancies, e.g., intimal sarcoma, the vast majority of bone and STS histotypes can arise in the oral and maxillofacial region, albeit frequency may vary. As anticipated, osteosarcoma was the predominant histopathologic diagnosis in our case series comprising slightly over 50% of all OSTJS in both children and adults, as well as 27.1% of the collective literature review diagnoses. In keeping with the current study, osteosarcoma was reported as the most common OSTJS in all previous case series that considered tumors of the gnathic bones in addition to intraoral STS [22–26]. Other non-osteosarcomatous malignancies occurring in the jawbones primarily included conventional and mesenchymal chondrosarcoma, Ewing sarcoma, *TFCP2::EWSR1*-rearranged rhabdomyosarcoma, and odontogenic neoplasms such as ameloblastic fibrosarcoma. Such lesions, however, are exceptionally rare and represent a mere fraction of OSTJS, collectively accounting for ~ 13% and 9% of all cases in our institutional and literature review analysis, respectively. Conversely, Kaposi sarcoma was identified as the most prevalent STS of the oral cavity, and 2nd overall most frequent OSTJS histotype in both analyses, encompassing 16-19% of all tumors. Similar findings regarding the incidence of Kaposi sarcoma in the oral cavity have been previously reported [26–28]. Notably, 61% (11 of 18) of Kaposi sarcoma diagnoses were rendered during the period 2000–2010, while only 3 (16.7%) new cases occurred in the last 7 years of our retrospective study. The observed decline in the frequency of oral Kaposi sarcoma mirrors the significantly decreasing incidence of AIDS-related Kaposi sarcoma in the United States in the last two decades, chiefly in white men 35–44 years of age, owing to the advent of cART as well as efficient preventative measures against transmission of HIV, e.g., PrEP medications [36–38]. Following Kaposi sarcoma, rhabdomyosarcoma, including embryonal, alveolar, and pleomorphic subtypes, and leiomyosarcoma were among the most frequently encountered oral STS. Notwithstanding their remarkably rare occurrence in the oral cavity, certain sarcoma variants demonstrate a strong site predilection. For instance, alveolar soft part sarcoma [39–42], *GLI1*-altered ST tumor [15, 43, 44], and epithelioid sarcoma [45–47], jointly comprising 1.5% (2 of 128) of OSTJS in our series, overtly favor the tongue compared to other intraoral anatomic sites.

Not surprisingly, greater than 95% of OSTJS in this institutional study presented as primary lesions and only 5 (3.9%) comprised metastases, namely 1 case each of leiomyosarcoma, epithelioid sarcoma, angiosarcoma, Ewing sarcoma, and high-grade sarcoma NOS. Overall, metastatic tumors to the oral soft tissues and gnathic bones are exceedingly rare constituting 1 − 1.5% of all intraoral malignancies [48–52]; the gingiva and tongue represent the most frequent sites for oral soft tissue metastases encompassing 54% and 23% of all cases, respectively [14, 48]. In a comprehensive review of 1,084 intraoral metastatic malignancies, sarcomas accounted for merely 0.7% of cases [48]. Since most sarcomatous tumors tend to spread hematogenously or via direct involvement of adjacent anatomic structures, rare metastatic involvement of the oral cavity is most likely due to pulmonary filtration or, alternatively, low affinity of mesenchymal malignancies for the jawbones and oral soft tissues, unlike carcinomas of renal, lung, breast and prostate primary origin [48]. A systematic meta-analysis of 123 metastatic oral sarcomas identified leiomyosarcoma (17%), angiosarcoma (16.3%) and osteosarcoma (14.6%) as the most common histopathologic subtypes affecting individuals with a mean age of 45.7 years and a peak incidence in the 7th decade of life, without any frank sex predilection (M: F = 1.12:1) [53]. Intraoral metastases are usually the sequela of disseminated sarcoma spread and prognosis, akin to metastatic carcinomas, remains dismal with a mean survival rate of ~ 8 months post-diagnosis [53].

Diagnosis of OSTJS in daily practice may be proven challenging owing to their comparative rarity, pronounced histologic diversity, and overlapping histomorphologic characteristics not just among sarcoma subtypes, but also OSTJS and non-mesenchymal mimics, e.g., spindle cell (sarcomatoid) SCC, melanoma, and follicular dendritic cell sarcoma. Adding to the level of diagnostic complexity, certain sarcomas that may occur in the oral and maxillofacial region can also show frank epithelial differentiation, for example synovial sarcoma [54, 55], or may demonstrate aberrant immunohistochemical expression of epithelial markers, i.e., pancytokeratins and EMA, such as frequently observed in epithelioid sarcoma [46, 47, 56, 57], epithelioid variant of angiosarcoma [58–60], adamantinoma-like Ewing sarcoma [61], and *TFCP2*-rearranged [62–69] and alveolar rhabdomyosarcomas [70]. Conversely, mesenchymal immunophenotypic properties have been occasionally reported in oral spindle cell SCC [71]. Cytogenetic analyses have significantly contributed to the elucidation of the molecular underpinnings and, therefore, better classification of soft tissue neoplasms, with many of these advances co-opted by immunohistochemistry for diagnostic applications [72–74]. Examples of antibodies serving as surrogate markers of sarcoma-specific genetic aberrations, e.g., fusions, amplifications, and point mutations, include SS18-SSX or SSX, TFE3, SMARCB1, BCOR, MDM2, DDIT3, and PAX3 [72–74]. Pertaining to OSTJS, irrefutably the diagnosis of jawbone osteosarcoma chiefly relies on appreciation of the microscopic and radiographic findings. Additionally, although not entirely specific, strong and diffuse, nuclear SATB2 immunostaining characterizes more than 90% of osteosarcomas [75–77], while confirmation of *MDM2* and *CDK4* amplification by FISH or immunohistochemistry may be diagnostically helpful in cases of parosteal or central osteosarcoma exhibiting low-grade cytomorphology, thus imitating benign fibro-osseous lesions of the jaws [78–82].

With the exception of most jawbone osteosarcoma cases, histopathologic diagnosis of other OSTJS typically necessitates immunohistochemical confirmation and/or identification of tumor-specific genetic abnormalities, as mentioned above. Special emphasis should be given to rhabdomyosarcoma harboring *TFCP2* rearrangement, a recently described, distinct and clinically aggressive OSTJS variant characterized by a biphasic epithelioid and spindle cell morphology, extensive bone and soft tissue destruction, and a strong predilection for young adults (median age: 25 years, range: 11–86 years) [15, 62–69]. Virtually all examples of *TFCP2*-rearranged rhabdomyosarcoma are diffusely positive for pancytokeratins, e.g., AE1/AE3, OSCAR, MNF116, and desmin, as well as myogenin and/or MYOD1, with MYOD1 showing higher sensitivity. Occasionally, ALK mRNA and protein overexpression has also been reported [15, 62–69]. In the absence of molecular analysis, strong cytokeratin immunoreactivity in such lesions comprises a major diagnostic pitfall with many *TFCP2*-rearranged rhabdomyosarcomas previously misinterpreted as spindle cell SCC. However, oral SCC would be exceptionally uncommon in the 2nd and 3rd decades in individuals without a predisposing genetic condition, such as Fanconi anemia, dyskeratosis congenita, or Li-Fraumeni syndrome.

## Conclusion

OSTJS represent a distinctly rare, histopathologically diverse, group of mesenchymal malignancies. In our series, most patients were adults in their 4th − 5th decade of life with a markedly broad age range and a slight male predilection. Osteosarcoma of the jaws and Kaposi sarcoma predominated among our cases, representing the most common intraosseous and soft tissue sarcoma subtype, respectively, in the oral cavity. Although the vast majority of cases comprised primary lesions, metastatic OSTJS were also identified. Diagnosis of OSTJS, akin to extraoral sarcomas, is based on appreciation of light microscopic findings in conjunction with ancillary immunohistochemistry and/or cytogenetic studies.

## Data Availability

No datasets were generated or analysed during the current study.
